# Transposition and fixation of lower pole crossing vessel in children with ureteropelvic junction obstruction: A STROBE-compliant study

**DOI:** 10.1097/MD.0000000000028235

**Published:** 2021-12-23

**Authors:** Vladimir V. Sizonov, Askhab H.-A. Shidaev, Johannes M. Mayr, Mikhail I. Kogan, Ilya M. Kagantsov, Vera V. Rostovskaya

**Affiliations:** a Regional Children's Clinical Hospital, Rostov-on-Don, Russia; b Rostov State Medical University, Department of Urology and Human Reproductive Health, Rostov-on-Don, Russia; c University Children's Hospital Basel, Spitalstrasse 33, Basel, Switzerland; d Research Institute of Surgery of Congenital and Hereditary Pathology Institute of Perinatology and Pediatrics, Federal State Budgetary Institution “Almazov National Medical Research Center”, Saint Petersburg, Russia; e First Moscow State Medical University, Department of Pediatric Surgery and Urology-Andrology, Moscow, Russia.

**Keywords:** Anderson-Hynes (dismembered) pyeloplasty, child, hydronephrosis, laparoscopy, lower pole crossing vessel, ureteropelvic junction obstruction, vessel transposition

## Abstract

Chapman and Hellstrom techniques are typically employed to transpose renal lower pole crossing vessels (LPCVs). Both procedures have certain limitations. We investigated the midterm outcomes in pediatric patients in whom LPCV-induced ureteropelvic junction obstruction was treated with either dismembered Anderson-Hynes pyeloplasty or upward transposition coupled with a new technique to fix the LPCV.

We retrospectively compared Anderson-Hynes pyeloplasty to the new technique in terms of outcome. LPCV transposition was considered feasible in patients in whom the diuretic loading test revealed a decrease in the pelvic volume after correction of vascular compression as well as absence of structural changes in the ureteropelvic junction (UPJ) and hemodynamic compromise of the lower renal pole. The fascial flap was passed below the LPCV to form a “hammock”. The free edge of the flap was sutured to its base.

Group 1 consisted of 102 (69.9%) patients (median age: 7.9 years) undergoing dismembered Anderson-Hynes pyeloplasty, while group 2 included 44 (30.1%) patients (median age: 8.4 years) treated with upward transposition and the new technique to fix the LPCV. No intra-operative complications or conversions occurred in either group. Redo-pyeloplasty was performed in 3 (2.9%) children of group 1 and 1 (2.3%) child of group 2. Renal ultrasonography conducted 12 months after surgery revealed similar anteroposterior diameters of the renal pelvis in groups 1 (7.9 ± 8.1 mm) and 2 (6.0 ± 2.9 mm). Patients in both groups showed a non-significant median increase in differential renal function at follow-up after at least 1 year after surgery (group 1: 36% [33.3; 40.5] vs 36.5% [35.3; 41.0]; group 2: 41% [37.5; 46.0] vs 43% [39; 46]).

In our patients, the new technique for laparoscopic or open fixation of the obstructing vessel after transposition was effective, reproducible, and devoid of limitations typical for the Chapman and Hellstrom techniques. We recommend Anderson-Hynes pyeloplasty in children with a history of hydronephrosis diagnosed antenatally, recurrent abdominal pain, intra-operative absence of peristalsis across the UPJ, high location of the UPJ at the renal pelvis, or intra-operative absence of volume reduction of the renal pelvis upon furosemide testing.

## 1 Introduction

An aberrant lower pole crossing vessel (LPCV) is the second most frequent cause of ureteropelvic junction obstruction (UPJO), occurring in 5.1% to 25% of children suffering from UPJO.^[1–3]^ In 1842, von Rokitansky^[4]^ first described UPJO caused by LPCV. Stewart^[5,6]^ was the first to describe treatment of UPJO by transposition of LPCV. The essence of the operation was to bring the poles of the kidney together, which led to the displacement of the LPCV above the zone of ureteropelvic junction (UPJ). Subsequently, Hellstrom et al^[7]^ described upward transition of the LCPV by suturing the adventitia of the crossing artery to the anterior pelvic wall. Brosig and Kollwitz^[8]^ introduced refinements of the original procedure in terms of hitching the LPCV and subsequent fixation to the front wall of the renal pelvis. Chapman^[9]^ postulated to place the LPCV in a tunnel formed by invagination of the anterior wall of the renal pelvis, thereby hitching the LPCV. The technical simplicity of upward transposition and fixation of LPCV compared to dismembered pyeloplasty proved to be a strong argument in favor of this surgical technique. This advantage gained particular relevance when laparoscopic access became feasible. The manual skills required to perform intracorporal ureteropelvic anastomosis are more demanding than those necessary for upward transposition of the LPCV.

The type of treatment of UPJO due to LPCV remains a matter of debate. Controversies concern the success of laparoscopic transposition of LPCV and lack of appropriate clinical and intra-operative criteria for selecting suitable patients for this type of surgery.

This retrospective study aimed to compare the midterm outcomes after either Anderson-Hynes pyeloplasty^[10,11]^ or upward transposition and fixation of the LPCV in patients suffering from LPCV-induced UPJO.

## 2 Materials and methods

### 2.1 Patients

Children suffering from UPJO caused by a LPCV treated either with dismembered Anderson-Hynes pyeloplasty (group 1) or upward transposition and the new technique to fix the LPCV (group 2) were included in this retrospective study. The surgical strategy and thus allocation of patients to the study groups were not influenced by the severity of the obstruction or clinical symptoms and findings.

LPCV transposition was considered feasible in patients in whom the diuretic test revealed a decrease in the pelvic volume after correction of vascular compression as well as absence of structural changes in the UPJ and hemodynamic compromise of the lower renal pole. The diuretic test was performed after ureterolysis and isolation of the LPCV. Furosemide was administered intravenously at a rate of 1 mg/kg body weight. We carried out visual control of the dynamics of renal pelvis dilatation. Transposition was considered feasible if the degree of pelvis expansion after diuretic test was less than before eliminating vascular compression.

In terms of treatment, we primarily considered transposition of LPCV rather than dismembered pyeloplasty in all patients with UPJO caused by LPCV. This intervention was considered feasible under the following intra-operative conditions:

- reduction of the renal pelvis volume upon intravenous administration of furosemide (1 mg/kg body weight) after transposition of LPCV
- absence of macroscopic abnormalities of UPJ
- UPJ located at the bottom of the renal pelvis
- restoration of active peristalsis at UPJ after altering the position of the LPCV
- normal color (marker of absent hemodynamic compromise) of the parenchyma of the lower kidney pole after transposition and fixation of LPCV.

Structural changes were visually detectable narrowing of the UPJ, high location of UPJ, and absence of UPJ expansion in response to diuretic test.

After performing vessel fixation, we assessed the color of the parenchyma of the lower pole of the ipsilateral kidney: appearance of a local (within the lower pole) change in color of renal parenchyma was considered as indicator of impaired renal blood supply inflicted by the altered position of the lower polar vessels, and we opted against transposition of the vessel in favor of the formation of antevasal anastomosis.

We did not intend to determine the cause of obstruction before pyeloplasty. However, the hypothesis of the presence of a LPCV in a patient as the cause of obstruction was formed, considering the following:

- the data of antenatal and postnatal imaging studies (in the absence of hydronephrosis in anamnesis, LPCV was assumed as the cause of obstruction),
- the patient's age (the older the child at the time of initial detection of obstruction, the more likely is a LPCV in our view),
- presence of clinical manifestations and, above all, pain (in the presence of recurrent lumbar pain, a LPCV was considered more likely),
- We did not undertake any additional efforts to visualize the LPCV.

### 2.2 Inclusion and exclusion criteria

We included all patients with UPJO caused by obstructing LPCV who were operated in our clinic between 2000 and 2020.

We excluded patients who suffered from UPJO caused by intrinsic stenosis of the UPJ, patients who did not undergo regular follow-up up to 1 year after the operation, and patients whose families opted against participation in this study.

### 2.3 Procedures

Hydronephrosis was confirmed by ultrasonographic imaging of the kidneys, diuretic dynamic renal scintigraphy, functional magnetic resonance urography, and spiral computed tomography. We conducted ultrasonographic imaging of the kidneys (emptied bladder, no water load) before and after surgery in all patients.

Indications for surgery were hydronephrosis grades III-IV according to the grading system of the Society of Fetal Urology, with a <40% decrease of differential renal function (DRF) on the affected side, recurrent upper urinary tract infection (UTI) despite antibiotic prophylaxis, abdominal/back pain, as well as a DRF decrease by more than 10% on the side of the lesion at follow-up.

The intraoperative conditions are described above.

### 2.4 Treatment allocation

Patients suffering from UPJO caused by LPCV were assigned to 2 groups. Group 1 consisted of 102 (69.9%) patients who underwent Anderson-Hynes dismembered pyeloplasty without reduction of the size of the renal pelvis, while group 2 contained 44 (30.1%) patients who were treated with upward transposition of LPCV according to the method described below.

### 2.5 Surgical technique of upward transposition and fixation of LPCV

**Figure 1:**
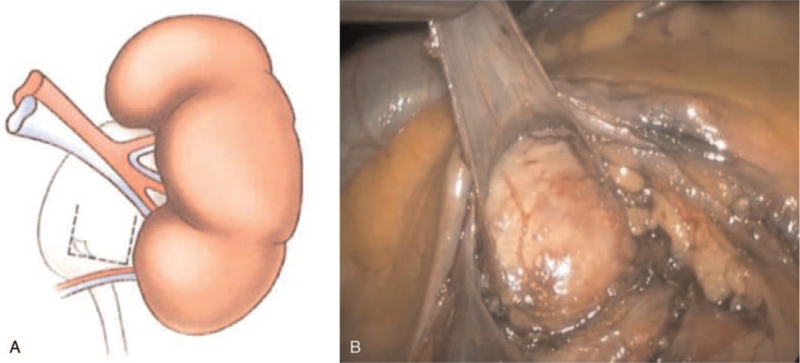
Mobilization of the fascial flap from the anterior wall of the renal pelvis. (A) Design of the flap (B) laparoscopic flap formation.

**Figure 2:**
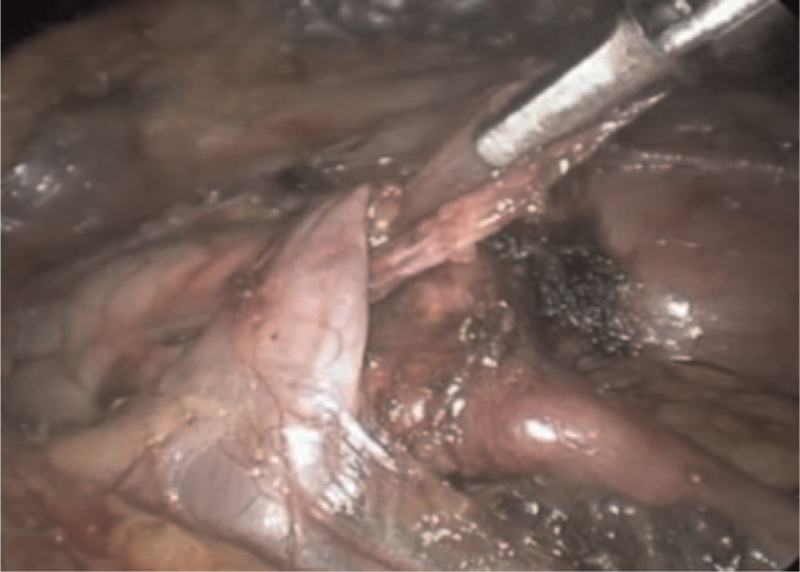
Positioning the fascial flap underneath the LPCV. LPCV = lower pole crossing vessel.

**Figure 3:**
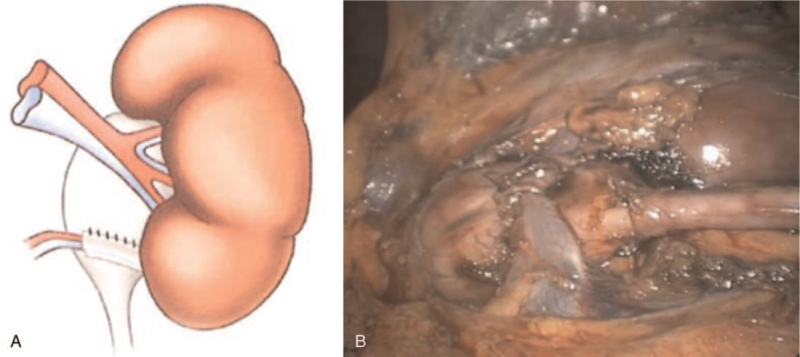
Fixation of the fascial flap (placed underneath the LPCV, flipped upwards, and sutured to the anterior wall of the renal pelvis). (A) Schematic drawing of the tunnel created for upward fixation of LPCV (B) laparoscopic view of tunnel containing the hitched LPCV. LPCV = lower pole crossing vessel.

After mobilizing the renal pelvis, UPJ, and LPCV, we formed a U-shaped flap from the fascia covering the anterior wall of the renal pelvis. We incised the fascia and created a horizontal incision (approx. 15 mm long) at the anterior surface of the renal pelvis 2 to 3 mm above the UPJ. Starting from the ends of the horizontal incision, the fascial flap was dissected upwards thus creating 2 parallel incisions in a cranial direction (15 mm long; targeted towards the main vessels of the kidney). The U-shaped fascial flap was created from the adventitial layer of the anterior wall of the renal pelvis (Fig. 1). Then, the LPCV was enveloped by the fascial flap, and the resulting sling was fixed cranially to the renal pelvis (Fig. 2). The free edge of the fascial flap was fixed to its base using interrupted sutures (Fig. 3), while ensuring the straight course of the vessels as well as avoiding tension and kinking of the crossing vessels.

#### 2.5.1 Criteria for inserting JJ stent or pyelostomy

We initially planned to complete all open dismembered pyeloplasties without draining the renal pelvis. Exceptions were patients who had a recent history of UTI. We placed a double J (JJ)-stent or pyelostomy if the tissue tension after resection of the pyelo-ureteral segment was adequate to form direct anastomosis but created tension in the area of the uretero-pelvic junction. Because no objective criteria for assessing the degree of tissue tension in the anastomosis area exist, this assessment was subjective and was based on the surgeon's experience.

We opted for JJ-stent or pyelostomy after anastomosis and diuretic test which was performed by intravenous administration of furosemide at a rate of 1 mg/kg body weight. Under conditions of diuretic load, even moderate extravasation of urine was considered as an indication for drainage. At the same time, we ensured that during the furosemide test, the diameter of the proximal ureter increased in response to passage of the urine bolus from the renal pelvis. Reduced filling and, as a consequence, insufficient expansion of the ureter below the anastomosis were regarded as an indication for the JJ-stent or pyelostomy placement. Choosing between placing JJ-stent vs pyelostomy was made by the surgeon and depended on the surgeon's experience and personal preference.

### 2.6 Follow-up of patients

In group 1 patients who were managed without stenting, we obtained first postoperative ultrasonographic images after 7 days. In patients of group 1 managed with JJ-stent or pyelostomy, ultrasonographic images were obtained after JJ-stent/pyelostomy removal. In group 2, follow-up ultrasonographic examination was performed on the 7th day after the operation and then after 3, 6, and 12 months.

Dynamic diuretic renal scintigraphy was carried out 1 year after surgery in all patients available for follow-up. We additionally assessed the number of UTI episodes occurring during the first year after surgery.

### 2.7 Treatment of recurrent or persisting UPJO

In patients with clinical symptoms, such as recurrent pain or UTI, and persisting dilation of the renal pelvis confirmed by ultrasonographic imaging of the kidneys, we performed diuretic dynamic renal scintigraphy before redo-pyeloplasty according to Anderson-Hynes.

### 2.8 Statistical analyses

Statistical analyses and data processing were carried out using the software STATISTICA 10 (version 10, StatSoft, Inc, Tulsa). Means, standard deviation, median, and interquartile range (lower and upper) were used as descriptive statistics for quantitative parameters. For quantitative data, we applied Mann–Whitney *U* test and Wilcoxon rank sum test. Qualitative parameters were analyzed by chi-square test, Yates correction, and Fischer exact test. A *P* value <.05 was considered statistically significant. We excluded patients lost to follow-up from all analyses.

### 2.9 Approval by ethics committee

The study was approved by the Local Independent Ethics Committee (LIEC) of the Federal State Budgetary Institution of Higher Education “Rostov State Medical University” (LIEC, protocol no. 13/2020).

## 3 Results

We retrospectively analyzed the medical records of 745 patients who had undergone surgery for UPJO between 2000 and 2020 at a single institution. We identified 146 patients whose UPJO was caused by LPCV and who were eligible for follow-up at least for 1 year after the operation. Group 1 consisted of 102 (69.9%) patients (median age: 7.9 years) undergoing dismembered Anderson-Hynes pyeloplasty, while group 2 included 44 (30.1%) patients (median age: 8.4 years) treated with upward transposition and the new technique to fix LPCV. In group 2, there were no cases of hydronephrosis diagnosed antenatally, and left-sided hydronephrosis was more common than in group 1. Recurrent abdominal/lumbar pain was reported more frequently in children of group 1. In terms of DRF, the 2 groups were comparable.

Table 1 shows the patient demographics and baseline characteristics.

**Table 1:** Demographics and baseline characteristics of patients in group 1 and group 2 (n = 146).

	Group 1 n = 102		Group 2 n = 44		
	Abs	%	Abs	%	*P* value
Boys, n	64	63	25	57	.5
Girls, n	38	37	19	43	
Median age (yrs) [Q1; Q3]	7 [4.2; 11.3]	–	6 [3.8; 12.0]	–	.36
Right, n	44	43	11	25	.038
Left, n	58	57	33	75	
Diagnosed antenatally, n	15	13.9	0	0	.006
Pre-operative pain episodes, n	84	77.7	4	8.5	<.001
Pre-operative UTI manifestation, n	8	7.4	2	4.2	.72
Pre-operative DRF, median [Q1; Q3]	36 [33; 40]	–	41 [37; 46]	–	<.001
Hydronephrosis grading III SFU	35	34	32	73	<.001
Hydronephrosis grading IV SFU	67	66	12	27	

DRF = differential renal function, Q = quartile, SFU = Society of Fetal Urology, UTI = urinary tract infection.

Surgical interventions were conducted by 3 different surgeons.

### 3.1 Reasons for choosing Anderson-Hynes pyeloplasty

Dismembered pyeloplasty was chosen because of structural UPI changes in 62 (60.8%) children, high location of UPJ in 42 (41.1%) patients, and a combination of the 2 factors in 28 (27.5%) patients. In 8 (7.8%) children in whom LPCV transposition failed to reduce pelvic volume and to restore peristalsis across the UPJ as evidenced by an intra-operative diuretic test, we switched to Anderson-Hynes pyeloplasty. Similarly, we changed to dismembering pyeloplasty in 4 (3.9%) patients in whom parenchyma of the lower pole had become discolored after vessel transposition.

### 3.2 Operative time and blood loss

Transposition and fixation of LPCV required significantly less operative time than dismembered pyeloplasty (*P* < .05). Laparoscopic transposition and fixation of LPCV required a median of 82 [61; 103] minutes, while the open-access procedure took a median of 73 [59; 88] minutes. In contrast, dismembered pyeloplasty required a median of 102.3 [75; 122] minutes and 92 [75; 110] minutes if performed laparoscopically or by open access, respectively. Median blood loss was 12 [9; 15] mL in group 1 and 9 [5; 13] mL in group 2 (*P* > .05) (Table 2).

**Table 2:** Operative details and drainage of renal pelvis and ureter in patients of groups 1 and 2 followed up for at least 1 year (n = 146).

	Group 1 n = 102		Group 2 n = 44		
	n	%	n	%	*P* value
Laparoscopic access	56	54.9	31	70.5	.08
Open access	46	45.1	13	29.5	
Drainless	46	45.1	44	100	<.001
Formation of pyelo-ureterostomy	24	23.5			
Formation of nephrostomy	11	10.8			
Insertion of ureteral stent	21	20.6			
Operating time (min), median [Q1; Q3] laparoscopic access	102 [75; 125]		82 [61; 105]		<.001
Operating time (min), median [Q1;Q3] open access	92 [75; 110]		73 [58; 89]		<.001
Blood loss, median [Q1;Q3]	12 mL [9; 15 mL]		9 mL [5; 13 mL]		.1
Success rate	99	97.1	43	97.7	.65

Q = quartile.

### 3.3 Success rate of operations

Success rates of the surgical interventions were similar in group 1 and group 2 (97.0% vs 97.7%; n.s.).

### 3.4 Operative complications

We observed no intra-operative complications in either group, and no conversions occurred when using laparoscopic access. Table 2 describes the surgical interventions as well as methods of drainage of renal pelvis and ureter applied in patients of group 1 and group 2 who were followed up for at least 1 year (n = 146).

In the postoperative period, 3 (2.9%) patients of group 1 who were managed without stents experienced urinary leakage through the retroperitoneal drain, necessitating insertion of a ureteral stent in 2 patients and percutaneous nephrostomy in 1 patient. After 6 months, these patients underwent redo-pyeloplasty. In group 2, 1 patient suffered recurrent UPJO 4 months after primary surgery. As evidenced by kidney sonography, dilation of the pelvicalyceal system remained at the pre-operative level, and periodic pain in the lumbar region persisted. During surgical revision, we noted structural changes in the pyelo-ureteral segment causing persistent obstruction. In this patient, we subsequently undertook pyeloplasty according to Anderson-Hynes.^[10,11]^

### 3.5 Follow-up of patients

During the first year after the operation, 9 patients were lost to follow-up. The final assessment took place at least 1 year after the operation in 102 (94.4%) patients of group 1 and 44 (93.6%) patients of group 2 (Table 2).

**Figure 4:**
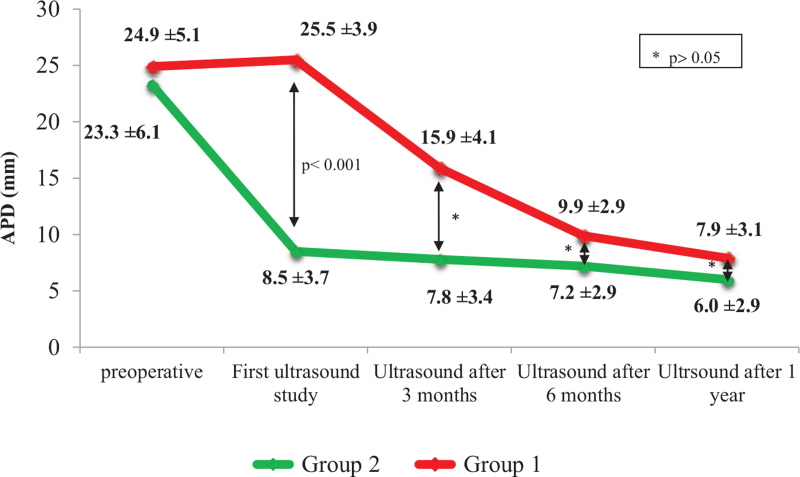
Ultrasonographic images of anteroposterior diameter (APD) of renal pelvis.

In group 1, mean pre-operative values of anteroposterior diameter (APD) of the pelvis did not differ significantly from those obtained immediately after surgery. At 3 months after the procedure, mean APD had significantly decreased, persisting until 1 year after surgery (*P* < .001; Fig. 4). In group 2, mean APD of the renal pelvis had decreased sharply by the time of the first postoperative ultrasonographic examination, already reaching values similar to those measured 1 year after surgery (Fig. 4; *P* < .001).

### 3.6 Comparison of APD and DRF 1 year postoperatively

At 6 months after surgery, mean values of APD were similar in both groups (9.9 ± 2.9 mm vs 7.2 ± 2.9 mm; n.s.). When compared to group 2, patients in group 1 exhibited a smaller mean reduction of APD between the first and second ultrasonography, but mean APD values measured 1 year after the operation did not differ significantly between the 2 groups (Fig. 4).

**Figure 5:**
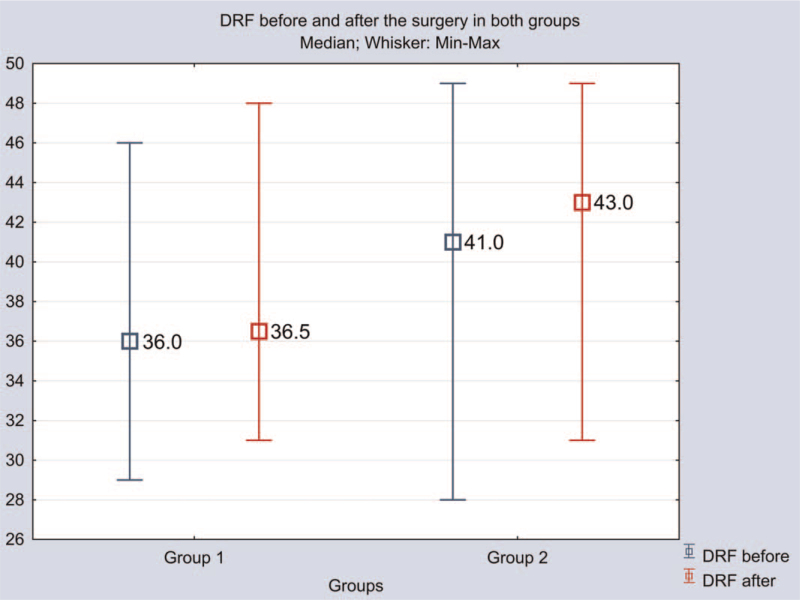
Differentiated renal function (DRF) dynamics in patients of groups 1 and 2.

As shown in Figure 5, patients in both groups showed a minimal balancing effect in DRF 1 year after surgery (group 1: 36% [33.3; 40.5] vs 36.5% [35.3; 41.0]; group 2: 41% [37.5; 46.0] vs 43% [39; 46]) (n.s.).

In all patients, urine tests were normalized, and bacteriuria was no longer present at 3 months after surgery.

## 4 Discussion

Careful pre-operative and intra-operative selection of patients with LPCV-induced UPJO who may be suitable for successful treatment by LPCV transposition and fixation is crucial. This requires careful scrutiny of patients’ medical records to eliminate any unnecessary risks.

In 75% to 100% of children with LPCV-induced UPJO, hydronephrosis is not detected antenatally,^[3,4,12,13]^ owing to the peculiarities of the kidney anatomy in children during their development. At birth, the kidney shows a U-like shape with a relatively short distance between kidney poles. At the same time, the vessels supplying the lower kidney pole take a course cranially to the UPJ thus not causing UPJ compression. During further growth of the kidney, the kidney poles divert thereby shifting the LPCV downwards, which may ultimately cause obstruction of the UPJ. Therefore, antenatal detection of hydronephrosis in the presence of LPCV is likely to be associated with intrinsic changes of UPJ causing obstruction.

We hypothesize that hydronephrosis identified antenatally is a contraindication for surgical upward transposition and fixation of the LPCV supplying the lower kidney pole. In addition, we noted that UPJ position at a higher point of the renal pelvis constitutes a contraindication for upward movement and fixation of LPCV in these patients.

A characteristic clinical manifestation of LPCV-induced UPJO is transient abdominal or lumbar pain. Recurrent pain has been described in 71.8% to 100% of children suffering from UPJO caused by LPCV.^[12–14]^ Among our patients who underwent upward transposition and fixation of the LPCV (group 2), recurrent abdominal or lumbar pain was reported in 4 of 44 (9.1%) patients. Incidences of recurrent pain in our study were considerably lower than those reported in the literature.^[12–14]^ However, in children with a history of recurrent abdominal/lumbar pain, Anderson-Hynes pyeloplasty should be the surgical technique of choice.

We applied 3 standard intra-operative criteria for assessing the feasibility of upward transposition of the LPCV, that is, reduction of the renal pelvis volume after releasing UPJ compression, spontaneous restoration of active peristalsis of the UPJ zone after upward transposition of the LPCV, and absence of macroscopic UPJ changes.^[5,15]^ Using these criteria, Polok et al opted for LPCV transposition in only 26 of 55 (47.3%) children suffering from UPJO caused by LPCV. Simforoosh et al^[16]^ performed upward movement and fixation of LPCV in 9 of 14 (64.3%) patients suffering from UPJO due to LPCV. Esposito et al^[12]^ argued that none of the known criteria for selecting patients for upward transposition and fixation of LPCV carries 100% accuracy in terms of excluding internal causes of UPJO. The authors proposed meticulous scrutiny of the patients’ medical history and thorough evaluation of intra-operative diuretic test results before opting for this type of surgery. In agreement with our results, Esposito et al^[12]^ achieved a success rate of ≥90% of the procedure when applying these selection criteria. Several groups reported successful treatment of UPJO by upward transposition and fixation of LPCV ranging from 96% to 100% when applying the patient selection criteria discussed above.^[15–21]^

Schneider et al^[22]^ conducted intra-operative diuretic testing in addition to the criteria mentioned above and opted for hitching of the LPCV in 8 of 19 (42%) patients with UPJO caused by LPCV. In all patients, hydronephrosis had resolved at postoperative follow-up; in 1 patient who was lost to follow-up, this could not be confirmed.^[22]^ Similarly, Parente et al^[23]^ extended the known criteria for patient selection by an intra-operative test with retrograde catheter insertion into the UPJ zone for balloon dilation in order to identify internal causes of UPJO. The authors stipulated that development of a “waist” in the UPJ zone after inflating the balloon under a pressure of 8 to 12 atm. is indicative for a combination of external and internal causes of obstruction. They recommended upward transposition of LPCV exclusively in UPJO children who did not exhibit the “waist” sign upon balloon dilation of the UPJ.^[23]^ The authors concluded that the additional endoscopic procedure did not prolong the time of intervention, nor did it affect the rate of postoperative complications or length of hospital stay (LOS).

In contrast, Villemagne et al^[21]^ warned that the typical clinical picture and careful use of intra-operative selection criteria do not definitively rule out an internal cause of UPJO in children suffering from LPCV-induced UPJO. The authors recommended selecting the type of surgery during the operation by relying solely on the experience of the surgeon.

According to the literature, the proportion of patients with UPJO due to LPCV treated successfully with upward transposition of LPCV ranges from 42% and 94%.^[5,14,16,22]^ The advantages of LPCV transposition over Anderson-Hynes pyeloplasty with resection of the UPJ in LPCV-induced UPJO include preservation of the integrity of the urinary tract as well as blood supply and innervation of the UPJ zone. In addition, time of surgery and LOS are significantly shorter than those associated with pyeloplasty. Nonetheless, generally accepted standards for selecting patients qualifying for LPCV transposition and fixation are still missing. Additional criteria should be defined to ensure the successful treatment of UPJO by LPCV transposition and fixation.

Our results indicate that patients whose UPJ is located at a higher point at the renal pelvis should not be treated with LPCV transposition and fixation. Similarly, patients with a history of recurrent abdominal pain or antenatal diagnosis of hydronephrosis are unsuited to LPCV transposition and fixation. Moreover, in patients in whom intraluminal stenosis cannot be excluded after intra-operative assessment of the UPJ status should undergo Anderson-Hynes pyeloplasty with antevasal ureteropelvic anastomosis.

While laparoscopic access is the method of choice for all children nowadays, this technique was largely reserved for older children in the past. During the first part of this study period, younger patients were mostly treated by the open access method until the laparoscopic technic had become sufficiently safe and effective also in smaller children.^[24]^

The Hellstrom^[7]^ and Chapman^[9]^ techniques have certain limitations. While the Hellstrom technique is limited by the lack of paravasal tissue required for reliable fixation of the LPCV to the pelvis after vessel mobilization, the Chapman technique can only be used if the pelvis is sufficiently dilated for invaginating the anterior wall and creating a tunnel for LPCV fixation. Our surgical technique proposed for LPCV transposition in UPJO does not have these limitations.

Patients treated by our surgical method (group 2) exhibited faster postoperative reduction of APD than those who underwent dismembering pyeloplasty (group 1). We hypothesize that in the absence of scars in the UPJ caused by dismembering pyeloplasty, faster restoration of normal peristalsis was achieved across the UPJ thus improving the transport of urine from the renal pelvis to the proximal ureter.

### 4.1 Study limitations and strengths

Due to the retrospective study design, limited number of patients, and follow-up interval of only 1 year, our results must be interpreted with caution. Allocation of patients to one of the 2 study groups was based on intra-operative decisions made by the surgeon in charge. Since 3 different surgeons were involved, our results cannot be generalized.

In group 1 patients, the most common indication for surgery was pain, whereas among group 2 patients, increased APD (determined ultrasonographically) and decreased DRF (measured by diuretic dynamic renal scintigraphy) were the most frequent indications for surgery. Although we did not use DRF or severity of symptoms as a selection criterion, group 1 patients showed more severe symptoms and lower DRF than group 2 patients.

Further limitations included heterogeneous patient characteristics, the surgeon-based choice of stent or drain insertion, and lack of randomization when choosing laparoscopic or open procedures.

The low rate of complications associated with the new laparoscopic technique for fixing the LPCV after upward transposition and low rate of conversions represent the strengths of our study.

## 5 Conclusion

The advantages of upward transposition and fixation of the LPCV compared to dismembered Anderson-Hynes pyeloplasty in patients suffering from UPJO caused by LPCV include maintaining the UPJ, unaltered blood supply to the proximal ureter, and intact innervation of the UPJ. Moreover, complications associated with drainage or ureteral stents are avoided, and time of surgery as well as LOS are markedly shorter. Upward transposition and fixation of LPCV is, however, feasible only in a selected group of patients. Patients with LPCV-induced UPJO in whom hydronephrosis was diagnosed postnatally and who do not have a history of recurrent abdominal or lumbar pain are considered possible candidates for this procedure. However, UPJ patency must be assessed intra-operatively before opting for this type of surgery. We consider an anatomic location of the UPJ at a higher point of the renal pelvis an additional contraindication for this technique.

Our technique of transposition and upward fixation of LPCV complements the existing range of surgical options used in LPCV-induced UPJO. Further prospective studies are required to confirm our findings.

## Acknowledgments

The authors thank Silvia M. Rogers, PhD, MEDIWRITE, Basel, for editing and correcting the manuscript.

## Author contributions

Vladimir V. Sizonov: research design, surgical interventions, data acquisition, data analysis, final approval of the manuscript.

Askhab H.-A. Shidaev: research design, surgical interventions, data acquisition, data analysis, manuscript preparation.

Johannes M. Mayr: manuscript preparation and revision.

Mikhail I. Kogan: manuscript preparation and final approval.

Ilya M. Kagantsov: data acquisition, surgical interventions.

Vera V. Rostovskaya: data acquisition, surgical interventions.

**Conceptualization:** Vladimir V. Sizonov, Askhab H.-A. Shidaev, Vera V. Rostovskaya.

**Data curation:** Vladimir V. Sizonov, Askhab H.-A. Shidaev, Ilya M. Kagantsov, Vera V. Rostovskaya.

**Formal analysis:** Vladimir V. Sizonov, Askhab H.-A. Shidaev, Ilya M. Kagantsov, Vera V. Rostovskaya.

**Investigation:** Vladimir V. Sizonov.

**Methodology:** Vladimir V. Sizonov.

**Supervision:** Vladimir V. Sizonov, Johannes Mayr, Mikhail I. Kogan, Vera V. Rostovskaya.

**Validation:** Vladimir V. Sizonov, Ilya M. Kagantsov, Vera V. Rostovskaya.

**Visualization:** Mikhail I. Kogan.

**Writing – original draft:** Askhab H.-A. Shidaev, Johannes Mayr, Mikhail I. Kogan.

**Writing – review & editing:** Johannes Mayr.
